# Supplementary material for: Gender difference in domain-specific quality of life measured by modified WHOQoL-BREF questionnaire and their associated factors among older adults in a rural district in Bangladesh
Source: PLoS One. 2025 Jan 7;20(1):e0317113. doi: 10.1371/journal.pone.0317113 (PMC11706451; doi:10.1371/journal.pone.0317113)
Supplement: S2 Data — (DOCX) [file pone.0317113.s002.docx]

Supplement 2: The original twenty-six items in the WHOQoL-BREF questionnaire, including domain names and item numbers (in brackets).

| Overall quality of life (1), Satisfaction of health (2) | | | |
| --- | --- | --- | --- |
| Physical domain:  Seven items  Pain (3)  Dependence on medical aids (4)  Energy (10)  Mobility (15)  Sleep and rest (16)**  Activities of daily living (17)  Work capacity (18)** | Psychological domain:  Six items  Positive feeling (5)  Personal belief (6)**  Concentration (7)  Bodily image (11)  Self‑esteem (19)  Negative feeling (26)** | Social domain: Three items  Personal relationship (20)  Sexual activity (21)  Social support (22) | Environmental domain:  Eight items  Security (8)  Physical environment (9)  Financial support (12)  Accessibility of information (13)  Leisure activity (14)  Home environment (23)**  Health care (24)**  Transport (25)** |

**items were excluded from the validated 19-item tool.
