# Supplementary material for: Gender difference in domain-specific quality of life measured by modified WHOQoL-BREF questionnaire and their associated factors among older adults in a rural district in Bangladesh
Source: PLoS One. 2025 Jan 7;20(1):e0317113. doi: 10.1371/journal.pone.0317113 (PMC11706451; doi:10.1371/journal.pone.0317113)
Supplement: Attachment — Submitted filename: PONE-D-24-44126_Tien Van Nguyen_Review.docx [file pone.0317113.s003.docx]

**General comments.**

Paper ID: **PONE-D-24-42119**

Paper Title: **Gender difference in domain-specific quality of life measured by WHOQoL-BREF questionnaire and their associated factors among older adults in a rural district in Bangladesh**

Reviewer Name: Tien Van Nguyen

Date: 30 October 2024

**Review**

***First impressions***

Overall, the research design is sound and the writing is well-structured. The background section is excessively lengthy and requires condensation. This study has provided valuable insights for policy makers and healthcare professionals to implement appropriate intervention programs to improve the QoL of older adults. The paper was well structured with the appropriate language used. The study results addressed the research question and objective. Notwithstanding, there are some specific issues within each section as detailed in the comments below.

***Introduction:*** Material hardship among older adults

The introduction was well-structured and provides comprehensive information regarding the paper, as well as was well organized and explained the importance of the study. Some points should be considered to improve the introduction:

The introduction is too long, the author should shorten it to suit the Objective of the research topic (About 2 papers A4).

Line 9 of Page 4: Authors should clarify before using abbreviations. For example: Quality of Life (QoL); And should be consistent in using QoL or QOL.

Line 18 of Page 4: Ref. 12 is too old. The author should consider citing appropriate references.

***Methodology***

- The author should review and unify the structure of the Research Methods section according to the guideline of Journal and consider merging the two contents ***Design and sampling & Sample size and statistical power***.
- Statistical analysis:

Authors should provide additional information:

+ Descriptive statistics: Applied to describe data about research subjects' information according to fall risk status: Number, rate (%); Mean (Standard Error) for variables following normal distribution; Median (range) for variables that do not follow a normal distribution.

+ Inferential statistics: statistically significant with: *** p ≤0.001; ** p ≤0.01; * p ≤ 0.05. Univariate and multivariate regression analysis to learn some factors related to the risk of falls in the elderly (statistically significant with 95%CI and *** p ≤0.001; ** p ≤0.01; * p ≤ 0.05).

+ The potential confounding variables to correct the results of the relevant Tables.

***Results and discussion***

Footnote of Table 5: Authors should add adjusted variables.

Furthermore, authors should explain the abbreviations in the tables in each Footnote at the end of each table (Table 3-5).

***Conclusion***

Acceptable.

***References, tables and figures***

The format of the references is inconsistent with and does not follow the journal's guidelines.

References should be cited at the end of each sentence and Authors should follow the Journal's reference citation requirements.

**Conclusion**

☒ Accept
