# Supplementary material for: Gender difference in domain-specific quality of life measured by modified WHOQoL-BREF questionnaire and their associated factors among older adults in a rural district in Bangladesh
Source: PLoS One. 2025 Jan 7;20(1):e0317113. doi: 10.1371/journal.pone.0317113 (PMC11706451; doi:10.1371/journal.pone.0317113)
Supplement: Attachment — Submitted filename: PONE-D-24-44126_revision letter R1_Islam.docx [file pone.0317113.s004.docx]

Title: Gender difference in domain-specific quality of life measured by modified WHOQoL-BREF questionnaire and their associated factors among older adults in a rural district in Bangladesh

Dear Editor,

Thank you for your extra ordinary feedback. Following your feedback, I would consider the manuscript has reached to an upper level. Thank you again.

Please see my responses in BLUE.

Best regards

Fakir M Amirul Islam

A/Professor, School of Health Science

Swinburne University of Technology.

Editorial comments:

Journal Requirements:

1. When submitting your revision, we need you to address these additional requirements.

Please ensure that your manuscript meets PLOS ONE's style requirements, including those for file naming. The PLOS ONE style templates can be found at 
<https://journals.plos.org/plosone/s/file?id=wjVg/PLOSOne_formatting_sample_main_body.pdf> and 
<https://journals.plos.org/plosone/s/file?id=ba62/PLOSOne_formatting_sample_title_authors_affiliations.pdf>

Response: The manuscript is now formatted according to PLOS One guidelines

2. When completing the data availability statement of the submission form, you indicated that you will make your data available on acceptance. We strongly recommend all authors decide on a data sharing plan before acceptance, as the process can be lengthy and hold up publication timelines. Please note that, though access restrictions are acceptable now, your entire data will need to be made freely accessible if your manuscript is accepted for publication. This policy applies to all data except where public deposition would breach compliance with the protocol approved by your research ethics board. If you are unable to adhere to our open data policy, please kindly revise your statement to explain your reasoning and we will seek the editor's input on an exemption. Please be assured that, once you have provided your new statement, the assessment of your exemption will not hold up the peer review process.

Response: Data are now made available.

3. Your ethics statement should only appear in the Methods section of your manuscript. If your ethics statement is written in any section besides the Methods, please move it to the Methods section and delete it from any other section. Please ensure that your ethics statement is included in your manuscript, as the ethics statement entered into the online submission form will not be published alongside your manuscript.

Response: It is now moved to methods section.

Additional Editor Comments:

Although reviewers have provided positive comments on your paper, I encourage the authors to address their comments in detail. Also, I suggest considering the following points to further enhance the paper.
Major points:
Introduction:
1. Focus on the scope of your works on QoL and how its distinction sexes. What social mechanisms are associated between factors and QoL in each sex group among elderly people are you seeking in your study.

Response: Thanks for a very useful suggestion. I have included the following statements:

Women have higher life expectancy than men, and they are susceptible to different disease patterns, which require different health-seeking behaviours than men [4]. For example, women need more care in residential environments, movement, connection, and emergency services than men. Men feel lonelier and more depressed than women [4, 5]. With people’s longer life expectancy, living arrangements also change. For example, the percentage of older adults living alone grows steadily and varies between genders. In 2020, approximately one-third of women aged 65 and older in the United States lived alone compared to 20% of older men [6]. In South Korea, the percentage of older adults living alone was 23.6% in 2017 (19.5% were men, 80.6% were women). The proportion of lonely deaths among older adults aged 60 years and older was reported to have increased significantly: 37.1% in 2017, 42.7% in 2019 and 47.5% in 2021[7]. Older men and women need different levels of community service.  For example, in a study among 1023 Korean older adults, 33.5% of men vs. 21.0% of women required support in preparing food, 48% of men vs. 63.1% of women needed maintenance support, 66.5% of men vs. 76.5% of women needed community support for any injury or accident. Gender and health shape aging decisively, thereby impacting the quality of life (QoL) in old age, and that is relevant in recognizing societal developments as well as in formulating adequate strategies for facing the challenges of aging according to their need [8].

2. What advantages of WHOQoL-BREF scale measures QoL among this population compared to other scales?

Response: Thanks. I have included the use of the modified version of WHOQoL-BRIEF scale (page 5 in track chances version, the reference numbers can be varied to this insertion). The justification for its use is as follows:

There are several tools for measuring QoL, but most of the research on QOL has focused on the impact of chronic diseases, such as cancer, stroke, diabetes and HIV/AIDS [18-21]. The WHO quality of life (WHOQOL) was developed using cross-cultural, multinational studies on the concept of QOL at the population level and proposed four fundamental domains: physical health, psychological health, social relationships, and environmental health [22-24]. This questionnaire has been used for extensive population studies [25], including its use in Bangladesh [26, 27]. Uddin and Islam [28] studied the psychometric properties of the WHOQOL-Brief 26-item questionnaire using Rasch analysis [29]. They proposed a modified version of 19-item tool, which was re-evaluated in a different sample [30] and proposed the retention of the 19-item tool for its use in the general population.

Please reconstruct the introduction to reflect for your research question and aims of study.

Response: I hope the introduction now reflect to the aims of my study.

Methods:
1. Data from surveys since 2017, please acknowledge the limitations of study.

Response: I have included the following limitation (at the end of the manuscript).

“data were also collected in 2017 which brings possibility of some social changes during the last 6-7 years”.

2. QoL: The QoL measure provides more robust justification for using this 50% cutoff point for QoL and cite relevant sources of evidence to support this cutoff point.

Response: I have included the following statements under “Measuring QoL using WHOQoL-BREF as the dependent variable”, page 12 (track changes version).

Rasch analysis [29] converts categorical values into quantitative logit scores based on a person’s ability and item difficulty with positive and negative values, a mean score of zero, and a standard deviation of 1. Then, the logit scores were converted on a 0 (worst QoL) to 100 (best QoL) score, 50 being the average QoL. This approach was proposed by Wright and Masters [60] in Rasch measurement theory.

3. Statistical analysis:
Back your study framework, you are trying to seek identifying the difference between 2 sexes (sample size estimation, page No.8), but your statistical analysis was not convinced (Table 3 to table 5) it is contradictory both using continuous variables and binary variables.
I invite authors to revisit data analysis to reframe for this again to have more comprehensive results, exploring interaction analysis between gender and predictors to gain deeper insights. The current approach to using subgroup analysis may not be sufficient to answer your research question.

Response: Thank you very much for your excellent suggestions. The interaction analysis has given greater insight into the data and improved the article significantly. Great suggestion!!

I have updated the sample size calculation. I have also provided interaction p values for each factor with gender.  I have incorporated the interaction in the results section, and the results are reflected in the discussion. However, I humbly disagree that the GLM and Odds ratio give contradictory results.  I want to keep both analyses.  The use of GLM and providing p values for interactions and p values for within total and by gender give adequate information to conclude that there are differences in factors associated with QoL, some equal and some differently.  The binary cut-off shows the proportion of people with below-average QoL in different domains and their related factors for both genders. Logistic regression analysis, in addition to the GLM, gives greater insight into the data.

**Sample size and statistical power**

The sample size of 573 males and 574 females was fixed for the current research, which was designed to investigate psychological distress and QoL among rural people. The study was previously described  [57, 58]. The current research indicates that the average quality of life (QoL) score in the psychological domain was 38.7 for males and 41.0 for females, with a pooled standard deviation of 13.9. The average value for the psychological domain was less than those of other domains. To calculate the sample size, the formula n = (Z_α/2_+Z_β_)2 *2*σ^2^ / d^2^ was used, where Z_α/2_ represents the critical value of the normal distribution at α/2 (e.g., for a confidence level of 95%, α is 0.05 and the critical value is 1.96), Z_β_ represents the critical value of the normal distribution at β (e.g., for a power of 80%, β is 0.2 and the critical value is 0.84), σ2 is the population variance, and d is the difference to detect. The given parameters produce a sample size of 572. Therefore, the current sample sizes of 573 males and 574 females were sufficient to detect a significant difference for all domains if the differences existed.  For comprehensive results, the QoL was dichotomized based on below-average QoL. In the psychological domain, the below-average QoL in females was 273 (47.6%) and in males 244 (42.6%), which yielded a risk ratio (95% confidence interval) of 1.12 (0.98, 1.27). For each cell frequency for the exposed and unexposed group 100, if the difference in the proportion of outcome is at least 14% (e.g., 50% vs. 36%), it produces a significant risk ratio.

**Statistical analysis**

Participants’ characteristics, including age, level of education, occupation, and socioeconomic status, were presented using descriptive statistics. Data were presented as numbers and percentages as all variables were categorical or were categorized into a few categories, such as age and level of education. A generalized linear model was used to present adjusted means and standard error of QoL for four domains by different factors. The QoL percentage on the linear scale from zero to 100 was divided into two categories: a percentage less than 50 was below average QoL, and 50 or above was equal or above average QoL. A binary logistic regression technique was used to estimate the odds ratio (OR) with 95% confidence intervals (CI) for each factor for below average QoL. The reference categories included age 60-69 years for the age group, no education level for the level of education, married for marital status, engaging in any income generation activities for occupation, living with family for living status, and own income for sources of income variables. The reference groups are shown in the results section. When an interaction between variables and gender was sought, the confounding variables were age, education, occupation, marital status, living status, and sources of income for model adjustment for the total sample, and to obtain the interaction p values. The variable gender was removed when stratification analyses for gender were obtained. RUMM 2030 [59] was used for Rasch analysis to compute the combined score from each domain. Statistical software SPSS (SPSS Inc, version 27) was used for descriptive data, GLM and logistic regression. Any results were statistically significant with p ≤0.001 for less than or equal to 0.1% significance level. Other than less than or equal to 0.1% significance level, the actual p values were presented. The odds ratio was significant if the confidence interval was less than 1.0 for an odds ratio less than and greater than or equal to one for an odds ratio greater than 1.0.

Results update:

Table 3 now has all interaction p values between factors and genders. For significant interactions, domain-specific QoL scores are presented graphically. The result section presents the following results related to interaction. The graphs are also presented.

“There were also significant interactions between different factors and gender in the association of the QoL domains.  (Table 3). In the case of the physical domain, the significant interaction was between the source of income and gender (<0.001) (Fig 1). In the case of the psychological domain, the significant interactions were education (p=0.002), occupation (p=0.006), and socioeconomic status (p=0.003) with gender (Figs 2.a) to 2.c)), respectively. For example, irrespective of education level, females’ QoL was low, whereas males with having SSC or above, being retired, engaged in any income activities, or having sufficient funds most of the time had significantly higher QoL in the psychological domain. In the case of the social domain, the significant interactions were age group (p=0.02), education (p=0.003), occupation (p=0.02), socioeconomic status (p<0.001), marital status (p<0.001) and income source (p=0.005) with gender (Figs 3.a) to 3.f)), respectively. For example, females aged 60-79 years had a mean QoL above 50% in the social domain, which drops to 41% after age 80. However, males’ social domain QoL was similar between ages 60 to 80 years. Females’ QoL in the social domain was the minimum even if they had an education of SSC or above, but males had the highest average QoL with SSC or above education.   In the case of the environmental domain, the significant interactions were education (p<0.001), occupation (p=0.003), socioeconomic status (p<0.001), and income source (p=0.005) with gender (Figs 4.a) to 4.d)), respectively.  Both males and females who were unable to work or engaged in any income activities had similar QoL in the environmental domain, but males who had SSC or above education had significantly higher QoL than females. There were also significant interactions between different factors and gender in the association of the QoL domains.  (Table 3). In the case of the physical domain, the significant interaction was between the source of income and gender (<0.001) (Fig 1). In the case of the psychological domain, the significant interactions were education (p=0.002), occupation (p=0.006), and socioeconomic status (p=0.003) with gender (Figs 2.a) to 2.c)), respectively. For example, irrespective of education level, females’ QoL was low, whereas males with having SSC or above, being retired, engaged in any income activities, or having sufficient funds most of the time had significantly higher QoL in the psychological domain. In the case of the social domain, the significant interactions were age group (p=0.02), education (p=0.003), occupation (p=0.02), socioeconomic status (p<0.001), marital status (p<0.001) and income source (p=0.005) with gender (Figs 3.a) to 3.f)), respectively. For example, females aged 60-79 years had a mean QoL above 50% in the social domain, which drops to 41% after age 80. However, males’ social domain QoL was similar between ages 60 to 80 years. Females’ QoL in the social domain was the minimum even if they had an education of SSC or above, but males had the highest average QoL with SSC or above education.   In the case of the environmental domain, the significant interactions were education (p<0.001), occupation (p=0.003), socioeconomic status (p<0.001), and income source (p=0.005) with gender (Figs 4.a) to 4.d)), respectively.  Both males and females who were unable to work or engaged in any income activities had similar QoL in the environmental domain, but males who had SSC or above education had significantly higher QoL than females.”

Discussion
4. It is lengthy, please concentrate on your main findings on how its distinct between sexes on QoL and its predictors. Offer specific recommendations based on your findings and future direction.

**Response:**  I have revised the discussion. I have included the following section before the strengths and limitations of the study.

Bangladesh has seen a spike in older adults over the years due to the rapid increase in life expectancy.   By 2050, people aged 60 and above are projected to constitute 22 per cent of the total population. Population ageing presents social, economic and cultural challenges to individuals, families, societies and the global community [75]. Although traditionally older people reside with their children and grandchildren, these traditional family care practices are no longer sustainable due to dynamic demographic and social changes that have transformed family structures and concepts, as well as vast migration from rural areas to cities and abroad. Thus, the trend of the number of people living alone is increasing. As a result, older adults, especially women and widows, economically poor individuals, and without engagement in any income generation activities have become more vulnerable and often lack adequate care and support [76, 77]. Loneliness is significantly associated with mental illnesses such as depressive symptoms and anxiety [78, 79]. Therefore, it is imperative to make appropriate strategies and policies and take various support programs, such as life skills education programs, focusing on older women to meet the challenges to improve health and well-being for ageing populations now and in the coming years.

**Reviewer #1:** The manuscript entitled “Gender difference in domain-specific quality of life measured by WHOQoL-BREF questionnaire and their associated factors among older adults in a rural district in Bangladesh” highlighted a significant correlation between engagement in income- generating activities and improved QoL in the social and environmental domains for elderly females in rural Bangladesh. This finding offers valuable insights for healthcare professionals seeking to improve the overall health and well-being of the elderly population.
However, the manuscript needs further improvement. Please see details in the specific comments below.
Specific comments
Major concern
1. In title of this manuscript: Author didnot use the original of WHOQoL-BREF questionnaire, then should you revise this title with “modified WHOQoL-BREF scale”?

Response: Thanks. It is done.

Gender difference in domain-specific quality of life measured by modified WHOQoL-BREF questionnaire and their associated factors among older adults in a rural district in Bangladesh

2. In the Methods section, the author should clarify whether the score of the modified WHOQoL-BREF (19 items) using Rasch analysis is equivalent to the original WHOQoL scale (26 items).

Response:

Response: The justification of the modified version of the WHOQoL-BREF 26-item is now presented in the background section.

Uddin and Islam [28] studied the psychometric properties of the WHOQOL-Brief 26-item questionnaire using Rasch analysis [29]. They proposed a modified version of 19-item tool, which was re-evaluated in a different sample [30] and proposed the retention of the 19-item tool for its use in the general population.

3. How to access SES variable in this manuscript is not appropriate for measuring of SES. Do authors have any reason to explain?

Response: Since there is no taxable income for the village people and also difficult to measure socio-economic status (SES) directly, a crude measure of SES was adapted in accordance with a similar study from Mongolia (Reference 61).  However, it is acknowledged that the measures of SES were not perfect.

The following limitation was presented

“Thirdly, the subjectivity in reporting the availability of funds in the last 12 months differs from the appropriate measure of SES”.

4. Authors showed in table 4 that among males, the prevalence of below average QoL in psychological domain odds ratio (OR) 1459774202.83, 95% confidence interval (CI)): 0, was higher among those who were unmarried or never married compared to those who married. Do you have any reason for this results? How is the goodness of fit in multivariable logistic regression which authors presented?

Response: Thanks for pointing out the reporting error.  One cell frequency was zero, which led to an infinitely large odds ratio and confidence interval.  I have removed the entry.

Minor comments
5. There are some minor writing issues in the manuscript.

Response: Thank you for your feedback. I have checked for grammatical errors, but I acknowledge that there may still be minor issues to address during the proofreading stage.

6. In Tables 2 and 3, the authors should provide the full name of the categorized SES and source of income variables, ensuring consistency with the methodology outlined on page 9 (independent variables).

Response: Thanks. Now the following is written in the text and the Tables.

The possible answers were: (i) insufficient funds for the whole year, (ii) insufficient funds for some of the time, (iii) neither deficit nor surplus (balance), and (iv) sufficient funds most of the time.

**General comments.**

Paper ID: **PONE-D-24-42119**

Paper Title: **Gender difference in domain-specific quality of life measured by WHOQoL-BREF questionnaire and their associated factors among older adults in a rural district in Bangladesh**

Reviewer Name: Tien Van Nguyen

Date: 30 October 2024

**Reviewer 2**

***First impressions***

Overall, the research design is sound and the writing is well-structured. The background section is excessively lengthy and requires condensation. This study has provided valuable insights for policy makers and healthcare professionals to implement appropriate intervention programs to improve the QoL of older adults. The paper was well structured with the appropriate language used. The study results addressed the research question and objective. Notwithstanding, there are some specific issues within each section as detailed in the comments below.

Response: Thank you very much for your very positive feedback

***Introduction:*** Material hardship among older adults

The introduction was well-structured and provides comprehensive information regarding the paper, as well as was well organized and explained the importance of the study. Some points should be considered to improve the introduction:

The introduction is too long, the author should shorten it to suit the Objective of the research topic (About 2 papers A4).

Response: Thanks. I have deleted some text and added some text explaining the rationale for using the WHOQoL questionnaire. The introduction is lengthier than your suggestions to make a cohesive flow and keep the background comprehensive. I hope you will accept it.

Line 9 of Page 4: Authors should clarify before using abbreviations. For example: Quality of Life (QoL); And should be consistent in using QoL or QOL.

Response: Thaks. Now, I have used QoL throughout.

Line 18 of Page 4: Ref. 12 is too old. The author should consider citing appropriate references.

Response: Thanks. I have updated the references by removing some old ones.

***Methodology***

- The author should review and unify the structure of the Research Methods section according to the guideline of Journal and consider merging the two contents ***Design and sampling & Sample size and statistical power***.

Response: Now the sub-title is: “***Study Design, s*ample size and statistical power”.**

- Statistical analysis:

Authors should provide additional information:

+ Descriptive statistics: Applied to describe data about research subjects' information according to fall risk status: Number, rate (%); Mean (Standard Error) for variables following normal distribution; Median (range) for variables that do not follow a normal distribution.

Response: In the analysis section, the following statement is included.

Data were presented as numbers and percentages as all variables were categorical or were categorized into a few categories, such as age and level of education.

+ Inferential statistics: statistically significant with: *** p ≤0.001; ** p ≤0.01; * p ≤ 0.05. Univariate and multivariate regression analysis to learn some factors related to the risk of falls in the elderly (statistically significant with 95%CI and *** p ≤0.001; ** p ≤0.01; * p ≤ 0.05).

Response: I have updated the section accordingly (Statistical analysis section).

Any results were statistically significant with p ≤0.001 for less than or equal to 0.1% significance level. Other than less than or equal to 0.1% significance level, the actual p values were presented. The odds ratio was significant if the confidence interval was less than 1.0 for an odds ratio less than 1.0 and greater than or equal to one for an odds ratio greater than 1.0.

+ The potential confounding variables to correct the results of the relevant Tables.

Response: When an interaction between variables and gender was sought, the confounding variables were age, education, occupation, marital status, living status, and sources of income for model adjustment for the total sample, and to obtain the interaction p values. The variable gender was removed when stratification analyses for gender were obtained (please see statistical analysis section and the relevant footnotes) .

***Results and discussion***

Footnote of Table 5: Authors should add adjusted variables.

Response: It is done.

Furthermore, authors should explain the abbreviations in the tables in each Footnote at the end of each table (Table 3-5).

Response: These are explained within the Tables.

***Conclusion***

Acceptable.

***References, tables and figures***

The format of the references is inconsistent with and does not follow the journal's guidelines.

References should be cited at the end of each sentence and Authors should follow the Journal's reference citation requirements.

Response: The references are fixed.

**Conclusion**

☒ Accept

**Response: Thanks.**

**Reviewer #3:** A very interesting study on quality of life. The gender aspect is a very interesting aspect to explore in this field and the findings from the study are noteworthy. Especially on the marital status of women and the income status of men.
But I feel that the author's Introduction is quite rambling, and needs to go directly to the quality of life and gender issues. The author should consider shortening this part.

Response: Thanks. I have deleted some text and added some text explaining the rationale for using the WHOQoL questionnaire. The introduction is lengthier than your suggestions to make a cohesive flow and keep the background comprehensive. I hope you will accept it.

Besides, the study was conducted in only one district so I am quite concerned about the representativeness of this district for the whole rural area of Bangladesh. Of course the author also mentioned "in a rural district" in the title and this is not a big problem for such a good study.

Response: Thanks.

The age group comparisons in the article are not appropriate as there are some comparisons with adult subjects while this study focused on the elderly. The author should replace the references appropriately.

Response: Thanks. I agree that the reference group 60-69 for 70-79 or 80+ is inappropriate as all are older adults (a homogenous group) and does not show any associations with any QoL domains, except marginally significant with the psychological domain (p=0.02).

The division of marriage and age groups seems inappropriate as the 60-69 group accounts for a large proportion. Age can be considered as a continuous variable in multivariate analysis.

Response: Many thanks.  I am sorry that I have not understood the relevance of this comment, as both age and marital status are independent variables.  Both variables are in the model only when the association of marital status with QoL domains is adjusted.  Other than this, there is no coexistence of these two variables.  Therefore, I am unable to understand the necessity of this analysis.  I hope you agree with my thoughts.

Interviewing on quality of life requires consistency among the interview team, the author needs to briefly supplement the interview process, interviewer selection, training and control of possible errors.

Response: Now the following information is added to make the data collection process more informative.

For data collection, first, the chief investigator at Swinburne University of Technology, in cooperation with the Organization for Rural Community Development (ORCD), a non-government organization in Bangladesh, recruited six data collectors. Second, all data collectors participated in an intensive two-day training program in Narail before they commenced the survey. The purpose of the training was to outline the study's rationale, procedures, and potential difficulties associated with data collection. Two interviewers were in one group for data collection, comprising three teams. Third, the data collectors contacted village leaders for their consent to collect data. Once they received the consent, they approached the prospective participants to explain the purpose of the study and to invite them to participate. Interviews were conducted upon their consent. One female was interviewed immediately after an interview of a male participant to maintain an approximately equal number of male and female participants. A pre-test of the questions in the final questionnaire was conducted by interviewing five lay people. Five women and men who were independent of the study team were interviewed using the questionnaire and were asked to assess comprehension, wording, and appropriateness. Five per cent of the entire data was entered twice by independent data entry operators to assure accuracy.

I do not have many comments on this manuscript and wish the authors to be approved for publication soon.

Thank you for your nice feedback!!
