# Supplementary material for: Gender difference in domain-specific quality of life measured by modified WHOQoL-BREF questionnaire and their associated factors among older adults in a rural district in Bangladesh
Source: PLoS One. 2025 Jan 7;20(1):e0317113. doi: 10.1371/journal.pone.0317113 (PMC11706451; doi:10.1371/journal.pone.0317113)
Supplement: Attachment — Submitted filename: PONE-D-24-44126_revision letter R2_Islam.docx [file pone.0317113.s005.docx]

Title: Gender difference in domain-specific quality of life measured by modified WHOQoL-BREF questionnaire and their associated factors among older adults in a rural district in Bangladesh

Dear Editor,

Thank you for your extra ordinary feedback. Following your feedback, I would consider the manuscript has reached to an upper level. Thank you again.

Please see my responses in BLUE.

Best regards

Fakir M Amirul Islam

A/Professor, School of Health Science

Swinburne University of Technology.

Editorial comments:

Introduction
Please narrow down the introduction, focusing on the research question. The current form is still quite lengthy. Cite only the most relevant references in the introduction. The current version with 52 citations is excessive.

Response: The introduction has been shortened from 902 words to 712 (~21% reduction).

The number of citations is 36 from 52. Some deleted references are below.

3.	Ageing and health. World Health Organisation 2024.

10.	Rautiainen LJ, Jansson AH, Knuutila M, Aalto UL, Kolster A, Kautiainen H, et al. Comparing Loneliness, Social Inactivity, and Social Isolation: Associations with Health-Related Quality of Life and Mortality among Home-Dwelling Older Adults. Gerontology. 2024:1-10. Epub 20240816. doi: 10.1159/000540345. PubMed PMID: 39154640.

15.	Richardson J, Sinha K, Iezzi A, Khan MA. Modelling utility weights for the Assessment of Quality of Life (AQoL)-8D. Qual Life Res. 2014;23(8):2395-404. doi: 10.1007/s11136-014-0686-8. PubMed PMID: WOS:000341820000024.

24.	Laskar MS, Rahaman MM, Akhter A, Sayed MHSU, Khan MH, Ahmad SA, et al. Quality of Life of Arsenicosis Patients in an Arsenic-Affected Rural Area in Bangladesh. Arch Environ Occup H. 2010;65(2):70-6. PubMed PMID: WOS:000278130800004.

28. Saleh F, Ara F, Mumu SJ, Hafez MA. Assessment of health-related quality of life of Bangladeshi patients with type 2 diabetes using the EQ-5D: a cross-sectional study. BMC Res Notes. 2015;8:497. Epub 20150929. doi: 10.1186/s13104-015-1453-9. PubMed PMID: 26420245; PubMed Central PMCID: PMCPMC4588249.

31.	Rahman E, Bardhan N, Curtin M, Islam MS, Patwary MFK, Kumar Das S. An assessment of disability and quality of life in people with spinal cord injury upon discharge from a Bangladesh rehabilitation unit. Spinal Cord. 2023;61(1):37-42. Epub 20221004. doi: 10.1038/s41393-022-00852-4. PubMed PMID: 36195670.

32.	Sarkar S, Rahman A, Islam N, Emran MA, Biswas J. Quality of life of stroke survivors in Bangladesh. SAGE Open Med. 2023;11:20503121231208604. Epub 20231108. doi: 10.1177/20503121231208604. PubMed PMID: 39139578; PubMed Central PMCID: PMCPMC11320675.

Cruz LN, Polanczyk CA, Camey SA, Hoffmann JF, Fleck MP. Quality of life in Brazil: normative values for the WHOQOL-bref in a southern general population sample. Qual Life Res. 2011;20(7):1123-9. Epub 20110129. doi: 10.1007/s11136-011-9845-3. PubMed PMID: 21279448.

Method
Outcome defines:
• “….Further, the revised model did not show any sign of local dependency. Two items are about general health and overall QoL. Each item scores 1 to 5 on a Likert scale where 1 represent”:
The new paragraph about the revised model and item scoring is confusing. Please rephrase it for better understanding.

Response: Thanks. I have clarified the paragraph as:

Different instruments, both generic and disease-specific, can measure QoL. WHOQoL-BREF [34] with 26 items, is a generic instrument to measure subjective QoL and is a known and acceptable cross-cultural comparison instrument available in more than 40 countries. Of 26 items, two are about general health and overall QoL, seven are about physical health, six are about psychological conditions, three are about social values, and eight are about surrounding environmental conditions. Each item scores 1 to 5 on a Likert scale where 1 represents (very dissatisfied/very poor), and 5 represents (Very satisfied). WHOQoL-BREF of 26 items was validated by Uddin and Islam [19, 21] and has proposed 19-item validated tools to measure QoL in rural Bangladesh. The modified 19-item tool [21] has five items for the physical domain, four items for the psychological domain, three items unchanged for the social domain and five items for the environmental domain. The modified 19-item tool showed adequate internal consistency, reliability, unidimensionality and freedom from differential item functioning for sex and age. Rasch analysis [20] converts categorical values into quantitative logit scores based on a person’s ability and item difficulty with positive and negative values, a mean score of zero, and a standard deviation of 1. Then, the logit scores were converted on a 0 (worst QoL) to 100 (best QoL) score, 50 being the average QoL. This approach was proposed by Wright and Masters [40] in Rasch measurement theory.

The original 26 items and domain-specific items used in this research are shown in Supplement 2.

• modified WHOQoL-BREF is not well described in this section; what we need to see in this section is briefly about the modified version, and you just need to cite previous work (PMC5588978).

Response: The modified WHOQoL-BREF has been described in some detail (response above). The protocol paper (reference below) has been shown as a reference for more information.

Uddin MN, Bhar S, Al Mahmud A, Islam FMA. Psychological distress and quality of life: rationale and protocol of a prospective cohort study in a rural district in Bangaladesh. BMJ open. 2017;7(9):e016745. Epub 20170901. doi: 10.1136/bmjopen-2017-016745. PubMed PMID: 28864700; PubMed Central PMCID: PMCPMC5588978.

Table 1 was not the result of this paper; please exclude it in this manuscript, or you can add it supplementary.

Response: I have included it as a supplement.

Supplement 2: The original twenty-six items in the WHOQoL-BREF questionnaire, including domain names and item numbers (in brackets).

Discussion
Please be cautious when comparing results to other studies. Since your revised 26-item questionnaire was applied only to the Bangladeshi population, direct comparisons may not be accurate. Consider discussing the potential discrepancies and limitations of such comparisons.
Shorten your discussion section

Response: I have included the statement as a limitation.

Finally, the QoL was measured using the modified version of WHOQoL-BREF[21] with 19 items. Although the modified version of WHOQoL-BREF was validated using Rasch analysis and proposed to be more suitable in measuring QoL in rural Bangladesh, the magnitude of average or below QoL was not directly comparable with those used the WHOQoL-BREF 26-item questionnaire.

Response: The discussion has been shortened from 1803 words to 1591 (~12% reduction).

Authorship: This is mostly your contribution; what others in the local or research team have not acknowledged and been authored in this work (PMC5588978, PMCPMC6451264)?

Response: Thank you very much for raising the error. I have acknowledged to Dr Nazim Uddin (PMC5588978, PMC6451264).

Prof. Sunil Bhar was involved in the supervision of Mohammad Nazim Uddin but did not have any role in conducting the research. A/Prof. Abdulla Al Mahmud had some involvement in PMC5588978 but not in the current research.
